# Unconventional animal models to study the role of telomeres in aging and longevity

**DOI:** 10.18699/vjgb-25-53

**Published:** 2025-07

**Authors:** E.V. Simoroz, J. Vasilevska, N.A. Arakelyan, A.D. Manakhov, E.I. Rogaev

**Affiliations:** Research Center for Genetics and Life Sciences, Sirius University of Science and Technology, Sirius Federal Territory, Krasnodar region, Russia; Research Center for Genetics and Life Sciences, Sirius University of Science and Technology, Sirius Federal Territory, Krasnodar region, Russia; Research Center for Genetics and Life Sciences, Sirius University of Science and Technology, Sirius Federal Territory, Krasnodar region, Russia; Research Center for Genetics and Life Sciences, Sirius University of Science and Technology, Sirius Federal Territory, Krasnodar region, Russia Vavilov Institute of General Genetics of the Russian Academy of Sciences, Moscow, Russia; Research Center for Genetics and Life Sciences, Sirius University of Science and Technology, Sirius Federal Territory, Krasnodar region, Russia Vavilov Institute of General Genetics of the Russian Academy of Sciences, Moscow, Russia

**Keywords:** telomere length, telomerase, aging, longevity, unconventional animal models, длина теломер, теломераза, старение, долголетие, неклассические модели животных

## Abstract

The progressive shortening of telomeres is significantly implicated in various cellular processes related to aging, including the limitation of cellular proliferative lifespan through the activation of DNA damage response pathways, ultimately leading to replicative senescence. Telomere shortening is considered an indicator of biological age rather than chronological age. The restoration of telomere length is mediated by the enzyme telomerase; however, it is crucial to maintain a balance in this process, as excessive telomerase activity and overly elongated chromosomes may increase the susceptibility of individuals to cancer. It has been proposed that variations in telomere length among individuals of the same chronological age may be associated with differences in potential lifespan. However, recent studies suggest that telomere length may serve only as a rough estimate of the aging process and is likely not a clinically relevant biomarker for age-related diseases or mortality risk. Furthermore, variations in telomere length are not solely determined by chronological age; rather, they are modulated by a multitude of factors, including genetic predispositions, environmental conditions, and heightened metabolic activities such as reproduction and body weight, which may lead to increased telomere attrition in certain species. It has been argued that traditional animal models, such as the mouse (Mus musculus) and the rat (Rattus norvegicus domestica), are suboptimal for investigating the relationship between telomere length and aging, as their lifespans and telomere lengths do not adequately reflect those of humans. Consequently, it is recommended to use long-lived species as they would provide a more appropriate framework for such research initiatives. This review aims to examine the correlation between telomere length and longevity in various non-traditional long-lived animal models, evaluating their suitability for investigating the molecular mechanisms underlying telomere attrition in the context of aging. Nevertheless, the question of whether telomere length is a causative factor or a consequence of longevity remains an area that necessitates further investigation.

## Abstract (ru)

Укорочение теломер играет важную роль в различных клеточных процессах, связанных со старением. Оно ограничивает продолжительность клеточной пролиферации и активирует механизмы, связанные с повреждением ДНК, что, в свою очередь, приводит к репликативному старению. Укорочение теломер отражает биологический, а не хронологический возраст. Механизм восстановления длины теломер осуществляется с помощью фермента теломеразы. Однако в этом процессе необходимо соблюдать баланс, поскольку избыточная активность теломеразы и чрезмерно длинные хромосомы могут привести к развитию онкологических заболеваний. Предполагается, что различия в потенциальной продолжительности жизни могут быть связаны с вариа циями в длине теломер у особей одного возраста. Тем не менее последние исследования показывают, что длина теломер может служить лишь приблизительной оценкой скорости старения и, вероятно, не является клинически значимым маркером риска возрастных заболеваний и смертности. Вариации в длине теломер часто обусловлены не только возрастом, но и генетическими изменениями, факторами окружающей среды, а также метаболически затратными процессами, такими как размножение и даже масса тела. Эти факторы могут способствовать ускоренной потере теломер у некоторых видов. Существует мнение, что для изучения роли длины теломер в контексте старения и долголетия классические модели, например мышь (Mus musculus) и крыса (Rattus norvegicus domestica), не оптимальны, поскольку продолжительность жизни этих животных и длина их теломер не сопоставимы с человеческими. Целесообразно использовать виды с более длительным жизненным циклом. В данном обзоре рассматривается степень корреляции между длиной теломер и долголетием в различных неклассических моделях долгоживущих животных, а также их пригодность для изучения молекулярных механизмов, приводящих к истощению теломер в контексте старения. Важно помнить, что вопрос о причинно-следственной связи длины теломер с продолжительностью жизни по-прежнему требует дальнейшего исследования.

## Introduction

Biological aging is characterized by a progressive decline in the functional capacities of an organism following the attainment of maturity, ultimately culminating in its demise. Substantial evidence indicates that telomere length may serve as a significant biomarker for aging and longevity. Telomeres are nucleoprotein structures that protect the ends of linear chromosomes from DNA degradation and are involved in repair processes, thereby playing a crucial role in maintaining chromosomal stability. With each cell division, telomeres undergo shortening due to the phenomenon known as the end-replication problem, particularly exacerbated by oxidative stress, which arises from an imbalance between the production of reactive oxygen species and the organism’s antioxidant defense mechanisms (Allsopp et al., 1995; Armstrong, Boonekamp, 2023).

It has been proposed that telomere length may significantly influence the allocation of resources between growth and reproduction, as well as the maintenance of the somatic state of cells (Young, 2018). This hypothesis is grounded in the aging theory articulated by T. Kirkwood (Kirkwood, 1977; Kirkwood, Rose, 1991), who posited that mortality associated with advanced age may result from an energy-conserving strategy designed to minimize the regulation of errors in somatic cells. Consequently, the metabolic costs associated with early reproduction may ultimately result in a depletion of energy resources essential for sustaining a stable somatic state, which could, in turn, expedite the aging process and increase mortality rates. Notably, in certain species, a negative correlation has been observed between telomere length and active reproduction.

**Fig. 1.**
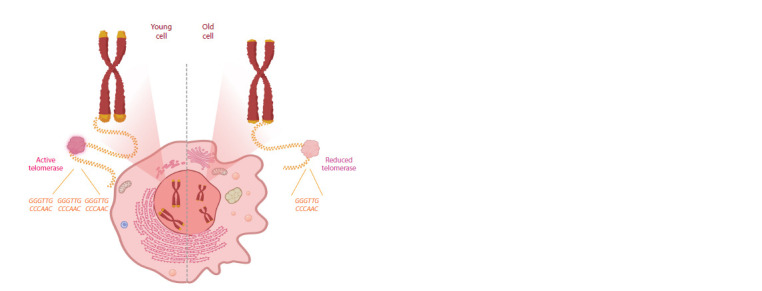
Variation in telomere length in relation to age and telomerase activity.

The principle of age-related telomere shortening is a complex phenomenon, and the biological mechanisms underlying this process remain poorly understood. Specifically, it is not yet clear whether telomeric aging functions as an analogue of the mitotic clock or serves as a biomarker of cellular stress (Koliada et al., 2015; Lin, Epel, 2022). Telomere attrition can be counteracted by telomerase, a specialized enzyme, the primary function of which is to extend telomeres. Telomerase is a ribonucleoprotein DNA polymerase composed of two subunits: telomerase RNA (TER) and telomerase reverse transcriptase (TERT). This enzyme facilitates the de novo addition of TTAGGG repeats to the terminal regions of chromosomes, thereby compensating for telomere loss. Figure 1 illustrates the variations in telomere length in relation to age and telomerase activity. Telomerase initiates its action by binding to the end of a telomere, with TER first interacting with the single-stranded DNA at the terminus of the chromosome. TERT utilizes the incorporated RNA as a template to synthesize new DNA repeats. This synthesis is typically mediated by reverse transcriptase, which generates a new segment of DNA by adding nucleotides to the single-stranded DNA of the telomere. The newly synthesized DNA fragment is complementary to the existing strand, as complementary nucleotides bind to the template, resulting in telomere elongation. Following the synthesis of several repeats, telomerase translocates along the telomere, allowing the enzyme to repeatedly add new nucleotides to the end of the chromosome. This process is repeated multiple times, significantly lengthening the telomere. After synthesizing a new stretch of DNA, the single-stranded DNA of the telomere can form a double helix by pairing with complementary strands. Additional enzymes, such as ligase and DNA polymerases, are also involved in this process to ensure proper telomere joining and completion (Nguyen, 2021).

There are two main telomere protection complexes: CST (Centriole- and Spindle-Associated Telomerase) and shelterin. These complexes can function in parallel in most mammals, including humans. The shelterin complex consists of six key components: TRF1 and TRF2 (Telomeric Repeat Binding Factor 1 and 2), TIN2 (TRF1-interacting protein), TPP1 (Telomeric Protein 1), POT1 (Protection of Telomeres 1), and RAP1 (Repressor-Activator Protein 1). The CST complex includes three components: Ctc1 (Cell Cycle Protein 1), Stn1 (Suppressor of Telomere Lengthening 1), and Ten1 (Telomere Length Maintenance 1). Both complexes play a crucial role in protecting and maintaining telomere structure and are also involved in regulating telomere length (Jenner et al., 2022; Zaug et al., 2022). Telomerase activity is notably elevated during the early stages of human fetal development; however, it is considerably restricted in the majority of normal adult cells. When telomeres attain critically short lengths, they initiate a persistent DNA damage response, which subsequently leads to various cellular processes, including cellular senescence and/or apoptosis. It also diminishes the capacity of stem cells to regenerate tissue. Furthermore, accelerated telomere shortening is a characteristic feature of age-related diseases which adversely impacts overall health and lifespan (Rossiello et al., 2022).

Research has indicated that telomere length may serve as a positive predictor of lifespan in humans. For instance, centenarians tend to possess longer telomeres, and studies have demonstrated that their offspring inherit this characteristic (Atzmon et al., 2010). Furthermore, healthy centenarians exhibit significantly longer telomeres compared to individuals afflicted with various diseases (Terry et al., 2008). Nevertheless, not all studies support this assertion, which raises questions regarding the reliability of telomere length as a biomarker for longevity and underscores the necessity for further investigation (Arai et al., 2015).

In many studies, traditional animal models, such as the mouse (Mus musculus) and the rat (Rattus norvegicus domestica), have been used to investigate the molecular mechanisms underlying aging and longevity (Sahm et al., 2018). Research involving genetically modified mice with hyperlong telomeres has demonstrated that these organisms exhibit reduced DNA damage as they age. Furthermore, these mice typically possess a lean body composition, lower cholesterol levels, enhanced glucose and insulin sensitivity, a decreased incidence of cancer, and an extended lifespan (Muñoz-Lorente et al., 2019). However, the applicability of these findings to humans is limited due to significant differences in telomere dynamics between the two species. For instance, the lifespan of mice is approximately 30 times shorter than that of humans, while their telomeres are 5 to 10 times longer and undergo shortening at a rate approximately 100 times faster. Additionally, there are notable disparities in the organization of repetitive elements and the shelterin complex within subtelomeric regions (Vera et al., 2012; Smoom et al., 2023). The complete absence of telomerase in mice results in a relatively weak phenotype over several generations, whereas heterozygosity for telomerase mutations in humans is sufficient to induce defects in organ regeneration and facilitate cancer progression (Calado, Dumitriu, 2013). Moreover, the majority of studies are conducted on specific inbred strains of rodents, such as C57BL/6 and BALB/c mice (Bernardes de Jesus et al., 2012). However, the lifespan observed in natural populations significantly surpasses that achieved in inbred strains, particularly due to anti-aging interventions conducted in vitro (Miller et al., 2002). This observation has prompted the consideration of alternative models with exceptionally long lifespans as potentially valuable for elucidating the mechanisms of telomere shortening and their viability as reliable biomarkers for aging and longevity.

This review aims to explore non-traditional long-lived animal models that may offer various advantages in supporting or challenging the role of telomeres as biomarkers that determine age and predict longevity (see the Table).

**Table 1.**
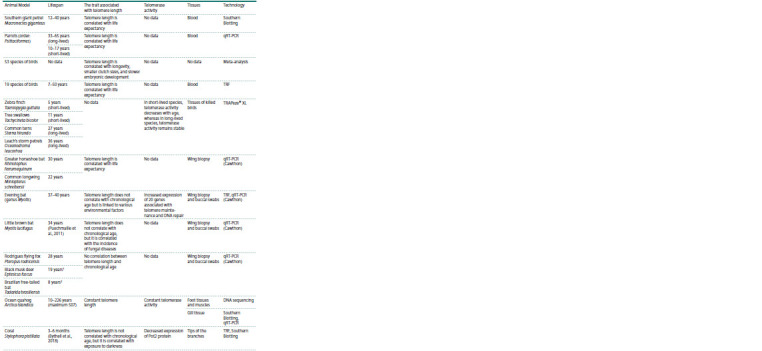
Relationship between telomere length, telomerase activity, and age in non-classical animal models

**Table 1end.**
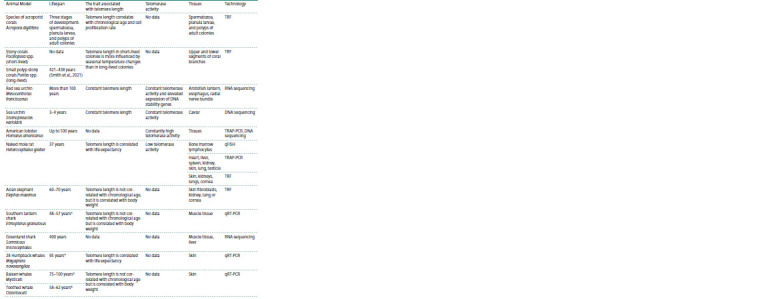
Table 1end. Note. TRF – restriction fragment assay; PCR – polymerase chain reaction; qFISH – quantitative fluorescence in situ hybridization; TRAPeze® XL – telomerase activity assay. Source of animal age data: 1 https://animaldiversity.org/accounts/Eptesicus_fuscus/#:~:text=Lifespan%2FLongevity,die%20in%20their%20first%20winter 2 https://animaldiversity.org/accounts/Tadarida_brasiliensis/ 3 https://fish.gov.au/docs/SharkReport/2023_FRDC_Etmopterus_granulosus%20_final.pdf 4 https://animaldiversity.org/accounts/Megaptera_novaeangliae/ 5 https://marilimitado.com/blog/fin-whale/ 6https://dlnr.hawaii.gov/dar/whales-and-dolphins/

## Birds

Birds serve as a distinctive model for investigating essential cellular mechanisms that are associated with extended lifespan. Despite their significant energetic expenditures throughout life, the majority of bird species can be classified as long-lived homeotherms exhibiting relatively slow aging processes. Notably, birds tend to have longer lifespans compared to mammals of equivalent size. Furthermore, critical aging processes in birds, such as cellular responses to oxidative stress and telomere dynamics, frequently exhibit similarities to those observed in mammals (Harper, Holmes, 2021).

A study conducted by C.G. Foote et al. examined telomere length as a potential indicator of the relationship between life history and fitness in southern giant petrels (Macronectes giganteus). In a cohort of adults aged between 12 and 40 years, no significant association was found between telomere length and age. However, individuals who died within an eight-year period following the measurement of telomere length exhibited significantly shorter telomeres compared to those who survived, irrespective of age or sex, which were not significant predictors of survival. These findings suggest that relatively short telomere length may serve as a biomarker for predicting life expectancy and may also indicate adult health (Foote et al., 2011). In a recent study, the telomere length in red blood cells and the markers of oxidative stress in plasma were examined in both long-lived and short-lived avian species belonging to the order Psittaciformes over a four-year duration. The findings indicated that long-lived birds, with lifespans ranging from 33 to 65 years, exhibited longer telomeres in comparison to their short-lived counterparts, which have lifespans of 10 to 17 years. However, it was noted that the long-lived birds experienced a higher rate of telomere shortening. Notably, the study established a significant correlation between the rate of telomere shortening and the levels of accumulated oxidative stress in short-lived birds. This correlation enhances the understanding of the underlying causes and dynamics associated with changes in telomere length (Domínguez-de-Barros et al., 2023, 2024).

Species exhibiting lower metabolic costs of reproduction at a young age are posited to develop more effective mechanisms for the maintenance and repair of somatic cells. This phenomenon may subsequently contribute to an increased potential lifespan and a deceleration of aging processes. In 2021, a phylogenetic meta-analysis encompassing data from 53 avian species was conducted to investigate the relationships among average telomere length in both chicks and adults, the average rate of change in telomere length throughout the lifespan, and various life history traits. The findings indicated that, irrespective of body size, longer-lived species characterized by smaller clutch sizes and slower embryonic growth rates demonstrate a reduced decline in telomere length over their lifespan (Criscuolo et al., 2021). Comparable results were observed in another study that analyzed telomere length across 19 bird species with lifespans ranging from 7 to 50 years. This study concluded that species with extended lifespans exhibited a slower decline in telomere length in comparison to those with shorter lifespans (Tricola et al., 2018).

Telomerase activity plays a crucial role in determining the rate of telomere attrition and is likely to have a direct influence on lifespan. A study conducted by M.F. Haussmann et al. examined telomerase activity in the bone marrow of two short-lived avian species: zebra finches (Taeniopygia guttata) and tree swallows (Tachycineta bicolor), which have maximum lifespans of 5 and 11 years, respectively. Additionally, two long-lived avian species were investigated: common terns (Sterna hirundo) and Leach’s storm petrels (Oceanodroma leucorhoa), which can live for 27 and 36 years, respectively. The findings indicated that the short-lived species exhibited high telomerase activity in nestlings; however, this activity declined significantly in both young and older adults. In contrast, the long-lived species maintained relatively high levels of telomerase activity in their bone marrow, which did not diminish with age (Haussmann et al., 2004).

In conclusion, the inherent anti-aging mechanisms observed in avian species render them more appropriate models for the study of longevity compared to short-lived laboratory rodents. Investigations focused on birds may ultimately contribute to the identification of therapeutic interventions for diseases related to human aging.

## Bats

Bats represent a distinctive subject for the investigation of aging and longevity. Similar to birds, they exhibit an atypical combination of small body size and extended lifespan relative to other mammals. For instance, individuals of the species Myotis brandtii can live for 40 years or more (Garg et al., 2023), whereas M. myotis has an average lifespan of approximately 37 years, Rhinolophus ferrumequinum about 30 years, and Miniopterus schreibersii around 22 years (Foley et al., 2018).

The existing literature on telomere length in bats presents conflicting findings. For instance, research indicates that telomeres shorten with age in species such as R. ferrumequinum and M. schreibersii, whereas this phenomenon is not observed in the genus Myotis, which is characterized by an extended lifespan (Gomes et al., 2011; Ineson et al., 2020). Furthermore, telomere shortening does not exhibit a correlation with age in species including Myotis lucifugus, Pteropus rodricensis, Eptesicus fuscus, and Tadarida brasiliensis. Notably, M. lucifugus individuals infected with the fungal disease known as white-nose syndrome (WNS) displayed significantly shorter telomeres compared to their uninfected counterparts (Ineson et al., 2020). These findings lend additional support to the hypothesis that environmental factors may exert an influence on telomere length, suggesting that telomere length is not invariably associated with the aging process.

The analysis of telomerase expression in wing fibroblasts and blood cells of M. myotis revealed that this enzyme is not expressed, suggesting the existence of alternative mechanisms that maintain telomere length. A more comprehensive investigation demonstrated a significant upregulation of 20 genes associated with telomere maintenance and DNA repair in Myotis bats when compared to other mammalian species. Notable among these genes are Atm and SETX, which have been identified as evolving under divergent selection in Myotis, alongside Abl1, Cct4, Dclre1a, Dot1l, Gnl3l, Mlh3, Mre11a, Parp1, Rad50, Rb1, Rfc3, Rpa1, Sde2, Ssb, Terf2ip, Wrap53, Wrn, and Xrcc5, all of which may play a role in maintaining genomic stability in bats. Furthermore, it was observed that variations in mean and minimum temperature, precipitation, and wind speed were significantly correlated with telomere length in bats (Foley et al., 2020). Consequently, telomere length may serve primarily as a biomarker for aging and longevity in specific contexts, although this does not appear to apply to the majority of bat species. Nonetheless, this model could be instrumental in examining the impact of environmental stressors on telomeres. Future research should explore whether variations in telomere length are linked to body size or survival strategies, such as hibernation or aestivation.

## Mollusks

Another noteworthy model for longevity studies is the mollusk. The North Atlantic oceanic quahog (Arctica islandica) is the most extensively researched long-lived bivalve, possessing the longest documented lifespan of at least 507 years. These organisms exhibit a remarkable tolerance to various environmental factors, including elevated salinity, temperature, and oxygen levels. It is important to highlight that the Icelandic population of A. islandica demonstrates an unusually high lifespan, whereas populations in the Baltic and White Seas have a maximum lifespan ranging from 30 to 50 years (Basova et al., 2012; Gruber et al., 2015).

The investigation of telomere length and telomerase activity in the longest-lived non-colonial organism, A. islandica, is crucial for comprehending the mechanisms underlying telomere length maintenance and its contribution to the organism’s exceptionally prolonged lifespan. An analysis of both short-lived and long-lived populations of young and old specimens (ranging from 10 to 226 years of age) revealed significant heterogeneity in telomere length among A. islandica. Notably, consistent telomerase activity and telomere length were observed across all age groups, with no correlation identified between these factors and population age or habitat. It is posited that stable telomere maintenance may play a role in the longevity of A. islandica; however, telomere dynamics alone do not account for its extraordinary lifespan (Gruber et al., 2014). Currently, the molecular mechanisms and potential mutations associated with this organism remain largely unknown, as the genome of A. islandica has yet to be published. Consequently, the specific factors that contribute to the longevity of this species are yet to be fully understood

## Corals

Due to their extended lifespans, corals represent a compelling yet underexplored model for investigating telomere responses to aging and environmental stressors. Recent radiocarbon dating of the deep-sea proteinaceous corals Gerardia sp. and Leiopathes sp. has revealed that these species exhibit radial growth rates ranging from 4 to 35 μm per year, with individual colonies possessing lifespans that extend to thousands of years. Notably, the oldest recorded individuals of Gerardia sp. and Leiopathes sp. were found to be 2,742 and 4,265 years old, respectively (Roark et al., 2009).

Research involving corals predominantly investigates the function of telomeres in response to stress. For instance, A. Rouan et al. (2022) analyzed telomere alterations in the symbiotic coral species Stylophora pistillata, which was subjected to a continuous dark environment for a duration of six months. This stressful condition led to a significant loss of symbiotic organisms. The study revealed that prolonged darkness was correlated with a reduction in telomere DNA length and a decrease in the expression of the Pot2 protein. In mammals, Pot2 forms a heterodimeric complex with Tpp1 and is essential for the recruitment of telomerase to telomeres. However, the authors did not establish connections between their findings and the concepts of aging or accelerated aging (Rouan et al., 2022). Additionally, another investigation explored the feasibility of using telomere length as a biomarker for estimating the age of colonial corals, specifically examining Acropora digitifera at three developmental stages: sperm, planula larvae, and polyps of adult colonies (Tsuta et al., 2014). The findings indicated that telomere length diminishes throughout coral development, with the highest values observed in spermatozoa, and the lowest in the polyps of adult colonies. It has been established that telomere length is affected not only by the chronological age of polyps but also by the rate of cell proliferation. Consequently, it can be inferred that attempts to accurately determine the age of corals based on telomere length may yield inconclusive results (Tsuta et al., 2014).

The long-term and short-term water temperature regimes are critical factors that influence intercolony variations in the Pacific Ocean. In this regard, telomere length was examined in short-lived, more stress-sensitive colonies of Pocillopora spp. and in long-lived, more stress-tolerant colonies of Porites spp. The findings indicated that telomere DNA length in short-lived colonies was significantly affected by seasonal temperature fluctuations. Conversely, telomere DNA length in long-lived colonies was not influenced by seasonal variations; rather, it was determined by historical thermal anomalies (Rouan et al., 2023).

In conclusion, the length of telomeres in corals is significantly influenced by environmental factors. The mechanisms involved in telomere maintenance are associated with the productivity of the organism, which is particularly relevant in light of the effects of climate change on overall health. Nevertheless, corals may not serve as optimal models for investigating the mechanisms of aging and lifespan, as their distinct environmental adaptations and slow metabolic rates could hinder the generalizability of findings to more complex multicellular organisms

## Sea urchins

Sea urchins serve as a compelling model organism for the investigation of developmental biology, longevity, and aging. Within this group, one can differentiate between shortlived species, such as Lytechinus variegatus and L. pictus, which have a lifespan that does not exceed four years, and long-lived species, including Mesocentrotus franciscanus, Strongylocentrotus purpuratus, Echinometra mathaei, and Stomopneustes variolaris, which can live for over 100 years and are recognized as some of the longest-lived organisms. The genomes of several species within this group have been sequenced and published, providing valuable resources for the study of genomic characteristics in relation to longevity (Sea Urchin Genome Sequencing Consortium et al., 2006; Sergiev et al., 2016; Polinski et al., 2024). Due to their capacity for indeterminate growth, sustained reproductive activity, and the absence of increased mortality with age, these organisms represent an ideal model for examining the phenomenon of negative aging (Ebert, 2019).

Research conducted within the established frameworks of aging theories, such as alterations in telomere length, has demonstrated that both short-lived and long-lived species of sea urchins exhibit minimal signs of aging. These marine organisms maintain consistent telomere length and telomerase activity. Furthermore, they preserve antioxidant and proteasome enzyme activities, and there is a negligible accumulation of oxidative cellular damage as they age. Their regenerative potential remains robust throughout their lifespan, irrespective of its length (Francis et al., 2006; Du et al., 2013). To investigate the mechanisms associated with longevity and aging in this model organism, a comparative analysis of gene expression in the radial nerve cord of M. franciscanus at various ages was conducted. This analysis identified over 4,000 differentially expressed genes that encompass a broad spectrum of cellular functions and molecular pathways, including neural function, metabolism, and the maintenance of DNA stability. Additionally, two genes, the expression levels of which increase with age, are implicated in the preservation of telomere length (Polinski et al., 2020).

A comparative study examined the amino acid sequence of the telomere-binding protein Pot1, which is crucial for the maintenance of telomere length through the regulation of telomerase-mediated elongation (Aramburu et al., 2020). In cell culture experiments, it was observed that mutations in the coding gene result in various telomere phenotypes, and the absence of this protein contributes to cellular aging (Zade, Khattar, 2023). Notably, the amino acid at position 198 of Pot1 exhibits variability across different species. In long-lived organisms, such as the red sea urchin and the bat (M. brandtii), this position is occupied by valine, whereas in short-lived species of sea urchins and bats, it is represented by threonine and serine, respectively. Interestingly, in humans and the long-lived naked mole rat, the amino acid at this position is isoleucine (Sergiev et al., 2016).

Although telomere length does not function as a reliable marker for longevity and aging in these models, it remains of significant interest for investigating the mechanisms underlying longevity and the preservation of DNA stability.

## Arthropods

Crustaceans represent a diverse group of arthropods characterized by significant variability in the size and structure of their genomes. For instance, the genus Homarus, which includes lobsters, is estimated to have a lifespan of up to 50 years in the wild and potentially up to 100 years in captivity. These organisms exhibit continuous growth throughout their lives, possess the ability to regenerate limbs even in advanced age, and older individuals may demonstrate greater fertility compared to their younger counterparts (Koopman et al., 2015; Bowden et al., 2020). A particularly noteworthy species within this group is the American lobster (H. americanus), which is distinguished by its remarkable longevity, potentially reaching 100 years. Lobsters serve as compelling subjects for research on longevity, aging, and telomerase function, as they may provide critical insights into the molecular mechanisms that underlie these exceptional longevity traits. Nevertheless, the available data on this model organism remain limited (Louzon et al., 2019).

Lobsters exhibit a slow aging process, making the analysis of telomerase activity in these organisms particularly intriguing. A study conducted in 1998 identified elevated telomerase activity across all organs of the lobster species H. americanus. The authors concluded that the activation of telomerase represents a conserved mechanism that aids in sustaining longterm cellular proliferative capacity and mitigating aging, not only in cellular models or during embryonic development but also in adult multicellular organisms (Klapper et al., 1998). Furthermore, the lobster genome was scrutinized for genes that contribute to DNA stability. Comparative analysis of expanded gene families in lobsters versus short-lived arthropods revealed the presence of Fancc and Ddb2, both of which play critical roles in maintaining genomic integrity (Polinski et al., 2021). Importantly, there is currently a lack of data regarding the relationship between telomere length and age in lobsters, underscoring the necessity for further investigation in this area.

## Naked mole-rat

The naked mole rat (Heterocephalus glaber) is a unique species notable for its remarkably extended lifespan, which can exceed 38 years. Traditional markers of aging, such as reduced reproductive capacity, neurodegenerative disorders, and cancer, are observed to manifest only minimally in this species (Yang et al., 2024). A study was conducted to examine telomere length across three age categories of naked mole rats: young, adult, and old. The results indicated that telomere length increased with age when compared to the young cohort; however, the authors acknowledged a limitation due to the small sample size. These findings support the hypothesis that telomeres are preserved in these animals as they age (Leonida et al., 2020). Additionally, naked mole rats exhibit low levels of telomerase activity (Seluanov et al., 2007), and a comparative analysis has revealed a negative correlation between telomerase expression levels and rodent size (Gomes et al., 2011). To date, the precise role of telomeres in the aging process remains unclear, suggesting that naked mole rats may offer valuable insights into the relationship between telomeres and aging

## Large animals (elephants, whales, and sharks)

The lifespan of large mammals is significantly influenced by their habitat, behavioral strategies, and physiological adaptations. For instance, forest elephants (Loxodonta cyclotis), which typically weigh between 2,000 and 2,500 kg, have an average lifespan of approximately 50 years. In contrast, African savannah elephants (L. africana), which weigh between 4,000 and 7,000 kg, and Asian elephants (Elephas maximus), weighing between 2,500 and 5,500 kg, can live for 60 to 70 years. These interspecies variations in longevity present a valuable model for investigating the molecular mechanisms of aging, including telomere dynamics (Crawley et al., 2017; Chusyd et al., 2021).

Comparative analyses of telomere length across species with differing lifespans indicate that initial telomere length may influence longevity. For instance, Asian elephants exhibit relatively longer telomeres at a young age when compared to Chihuahuas, a small dog breed that typically weighs between 1 and 3 kg and has a considerably shorter lifespan. Despite this initial advantage in telomere length, both elephants and Chihuahuas demonstrate similar rates of telomere shortening over time. This observation implies that while initial telomere length may partially contribute to determining maximum lifespan, the process of telomere shortening with age appears to be a relatively conserved trait across species.

An important factor associated with interspecies variations in longevity is the activity of telomerase and its correlation with body size. Research indicates that the suppression of telomerase activity in the somatic cells of larger mammals may represent an evolutionary adaptation aimed at decreasing the incidence of cancers commonly observed in organisms with greater body mass. Consequently, the restriction of telomerase activity and the presence of shorter telomeres in large, longlived species seem to function as an additional barrier against tumorigenesis (Buddhachat et al., 2017).

The lifespan of large mammals is intricately linked not only to their terrestrial counterparts but also to those inhabiting marine environments. Among cetaceans (Cetacea), which encompass the parvorders of baleen whales (Mysticeti) and toothed whales (Odontoceti), a considerable variation in longevity is observed. Numerous whale species, characterized by substantial body mass – ranging from several tons in dolphins to tens and even hundreds of tons in the largest species – exhibit remarkable lifespans. For instance, the bowhead whale (Balaena mysticetus), belonging to the family Balaenidae, is documented to live for over 200 years, thereby earning its status as one of the longest-lived mammals on Earth (Buddhachat et al., 2021; Lagunas-Rangel, 2021).

In contrast to several terrestrial species, for which a correlation between initial telomere length and potential lifespan has been established, the data concerning cetaceans remain more ambiguous. A study using quantitative PCR on skin samples from 28 humpback whales (Megaptera novaeangliae) aged 0 to 26 years revealed a statistically significant correlation between telomere length and age. However, the considerable variability observed among individuals of the same age suggests that telomere length cannot be regarded as a reliable indicator for determining age in free-swimming whales. This variability may be attributed to both methodological factors, such as measurement accuracy, and biological influences, including hereditary traits, adaptations to unpredictable environmental conditions, and stochastic processes related to resource allocation (Olsen et al., 2014).

Additional comparative studies encompassing 23 marine mammal species, which include four Mysticeti and 19 Odontoceti species, revealed no significant association between relative telomere length and maximum lifespan. Statistical analyses indicated that longevity exhibited a stronger correlation with body size; specifically, adult mass and length were identified as robust predictors, whereas relative telomere length did not demonstrate a significant association (Buddhachat et al., 2021).

Another marine species notable for its exceptional lifespan is the shark; however, determining their age can be particularly challenging. For example, the Greenland shark (Somniosus microcephalus) has recently been identified as the longest-living vertebrate on Earth. Despite this recognition, many aspects of its biology, physiology, and ecology remain insufficiently understood. This species can live for nearly 400 years and reaches sexual maturity at approximately 150 years of age, with weights ranging from 700 to 1,000 kilograms (Nielsen et al., 2016). Recent studies involving samples from Greenland sharks have enabled researchers to analyze RNA and identify a highly expressed long interspersed nuclear element-like (LINE-like) transcript (Bartas et al., 2023). It has been suggested that this transcript may be linked to an increased lifespan and enhanced resistance to age-related diseases. The authors of the study hypothesize that this factor could contribute to improved telomere maintenance. However, there is currently no scientific evidence to support this hypothesis, nor are there any data regarding telomere length in these animals (Bartas et al., 2023).

A separate study focusing on other shark species, such as the southern lantern shark (Etmopterus granulosus), examined the relative telomere length in relation to age, which in this case was assessed based on body mass (Nehmens et al., 2021). The findings indicated that telomeres in this species do shorten in accordance with size; however, it remains uncertain whether age directly influences telomere length in this context (Nehmens et al., 2021).

In conclusion, although the current body of research is insufficient to reach definitive conclusions, it can be suggested that large body size is a significant factor for the organisms discussed. This phenomenon may reflect a combination of evolutionary and physiological strategies that are aimed at maintaining genomic stability, regulating cell division, and reducing the risk of cancer.

## Conclusion

**Fig. 2.**
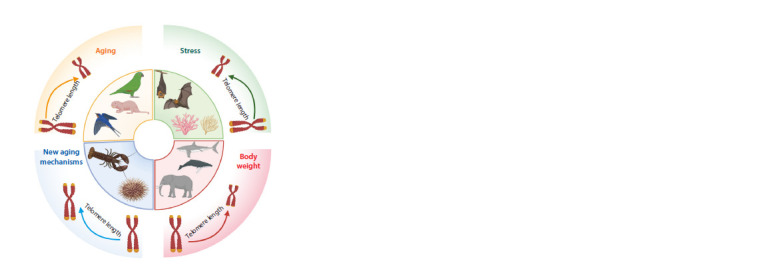
Factors affecting telomere length in various long-lived organisms

In this review, we summarize the distinctive characteristics of various alternative animal models that exhibit delayed and accelerated aging phenotypes. The species examined are notable for their exceptional longevity, significant regenerative capabilities, or resistance to age-related diseases. However, the findings from studies investigating the relationship between telomere length and age-related diseases, as well as lifespan, remain inconsistent and contradictory. In this context, birds and naked mole rats appear to be the most appropriate models for studying the mechanisms of telomere shortening in relation to aging and longevity, while bats and corals are more suitable for analyzing the effects of stressors on telomere length. Large, long-lived animals such as elephants, whales, and sharks demonstrate a correlation between telomere length and body mass. Sea urchins and lobsters are particularly intriguing for exploring alternative aging mechanisms that have yet to be identified (Fig. 2).

It is evident that all the aforementioned models possess unique mechanisms for life extension and telomere length maintenance that lack analogs in traditional model organisms. Nevertheless, the extent to which insights gained from longlived model organisms regarding aging and longevity can be applied to humans to promote a longer and healthier life remains uncertain. Therefore, optimizing the application of these models in applied research is crucial. Species exhibiting constant telomere length and active telomerase, such as Arctica islandica, Mesocentrotus franciscanus, and Homarus americanus, are particularly well-suited for in-depth investigations into the molecular mechanisms underlying extreme longevity and the identification of potential geroprotective targets. Organisms, in which telomere length varies significantly due to external factors, such as bats of the genus Myotis and corals of the genus Pocillopora, are appropriate for assessing the impact of environmental stressors, diseases, or habitat quality on aging processes. A combined study of sea urchins (e. g., Strongylocentrotus variolaris and Mesocentrotus franciscanus) or corals (e. g., Pocillopora spp. and Porites spp.) will facilitate the identification of factors influencing telomere dynamics across different lifespan scenarios. Large-scale models, including elephants, sharks, and whales, exhibit a correlation between telomere length, body mass, and lifespan, which may be beneficial for developing biomarkers of population health in natural settings. Data obtained from species with notable resistance to age-related pathologies, such as naked mole rats and Myotis bats, can be extrapolated to identify new therapeutic targets for age-related diseases in humans.

Thus, the use of alternative species with diverse aging strategies and unique telomere maintenance mechanisms not only broadens the scope for testing the hypothesis of telomeres as a universal biomarker of aging but also enables a more objective evaluation of their contributions to longevity and age-related pathologies. Incorporating these extreme models alongside classical organisms enhances our understanding of the fundamental mechanisms of longevity and opens new strategies for applying the knowledge acquired in medicine, ecology, and the conservation of endangered species, potentially aiding in the extension of the active and healthy lifespan of humans.

## Conflict of interest

The authors declare no conflict of interest.
